# People-centred science: strengthening the practice of health policy and systems research

**DOI:** 10.1186/1478-4505-12-19

**Published:** 2014-04-17

**Authors:** Kabir Sheikh, Asha George, Lucy Gilson

**Affiliations:** 1 Public Health Foundation of India, ISID Campus, 4 Institutional Area, Vasant Kunj, New Delhi 110070, India; 2 Johns Hopkins University Bloomberg School of Public Health, 615 North Wolfe Street, Suite E-8146, Baltimore, MD 21205, USA; 3 Department Global Health and Development, London School of Hygiene and Tropical Medicine, Health Policy and Systems Division, School of Public Health and Family Medicine, University of Cape Town, Health Economics and Systems Analysis Group, Private Bag X3, Rondebosch 7701, South Africa

**Keywords:** Context, Dialogue, Health policy and systems research, Health systems research, Knowledge translation, People-centred health systems, Reflexivity, Research practice, Social construction

## Abstract

Health policy and systems research (HPSR) is a transdisciplinary field of global importance, with its own emerging standards for creating, evaluating, and utilizing knowledge, and distinguished by a particular orientation towards influencing policy and wider action to strengthen health systems. In this commentary, we argue that the ability of the HPSR field to influence real world change hinges on its becoming more people-centred. We see people-centredness as recognizing the field of enquiry as one of social construction, requiring those conducting HPSR to locate their own position in the system, and conduct and publish research in a manner that foregrounds human agency attributes and values, and is acutely attentive to policy context. Change occurs at many layers of a health system, shaped by social, political, and economic forces, and brought about by different groups of people who make up the system, including service users and communities. The seeds of transformative practice in HPSR lie in amplifying the breadth and depth of dialogue across health system actors in the conduct of research – recognizing that these actors are all generators, sources, and users of knowledge about the system. While building such a dialogic practice, those conducting HPSR must strive to protect the autonomy and integrity of their ideas and actions, and also clearly explain their own positions and the value-basis of their work. We conclude with a set of questions that health policy and systems researchers may wish to consider in making their practice more people-centred, and hence more oriented toward real-world change.

## Health policy and systems research: a change-generating field

Health policy and systems research (HPSR) is widely recognised as a critical element of the action needed to achieve the health-focused Millennium Development Goals, strengthen primary health care, and promote universal health coverage [1]. Focussed on generating knowledge on how best to organize collective responses to promoting health and tackling disease over time, this research field studies international, national, subnational, and local health systems and their interlinkages, as well as policies made and implemented at all levels of the health system. In 2012, the successful 2nd Global Health Systems Research Symposium, and the formation of a Health Systems Research society, drew a large community of interested groups together not only to examine the available research knowledge but also to work towards developing and strengthening the practice and approach of the research conducted. Publication of the 2012 WHO HPSR strategy also sought to contribute to this latter goal by supporting the development of an identity for the field of work, with ‘*its own standards for evaluating evidence, assembling knowledge, and translating it into recommendations that decision makers in the health system can comprehend, trust, and implement*’ [2], p. 15. In 2014, this research and policy community will meet in South Africa to discuss ‘The Science and Practice of People-centred Health Systems’, the theme of this year’s Global Symposium on Health Systems Research.

There is consensus on some key features of HPSR [3,4]. Focus areas within it include the performance of health systems and their subcomponents (resources, organizations, and services), as well as consideration of how links among the subcomponents shape performance, what forces influence those links, and how to strengthen health system performance over time. HPSR is also recognized as a hybrid, or ‘trans-disciplinary’ field, drawing on different disciplinary traditions and methodological approaches, and with links back to the older field of health services research. Most critically, HPSR is applied research, undertaken with an orientation towards influencing policy and wider action to improve the performance of health systems, in the short or long term.

However, as those who work in this field come from different knowledge traditions, these differences continue to influence how they view health systems and how they approach knowledge generation and evaluation. In line with prior arguments [5-7], we seek in this commentary to elaborate directions for the practice of HPSR founded on the particular understanding that i) health systems are social and political constructs that, as part of the fabric of any society, provide vital opportunities for tackling social injustice; ii) human agency, in interaction with broader societal structures, fundamentally shapes health systems; and iii) social science perspectives and approaches offer particular value to this area of trans-disciplinary research. As authors, we come from eclectic backgrounds in public health, health policy studies, health economics, international relations, development studies, and medicine, with experience of HPSR mostly from India and sub-Saharan Africa. From these various bases, we argue that the potential of HPSR to achieve health system change hinges on it becoming more people-centred in how it is conceived, conducted, and utilized.

System change begins and ends with people because people, operating in various roles, ultimately make up any system and fundamentally shape how it works. Acknowledging that health systems are constituted by people and operate in social, political, and economic contexts defined by people and groups with varying interests and values, opens up a panoply of opportunities to influence and change them. It also requires researchers to acknowledge and address questions around their own role and power as actors in the same system.

The following sections both unpack our foundational understanding and outline directions for the emergence of such a people-centric practice of HPSR. Using three brief case studies from our own work, we consider how health policy and systems are conceptualised (seeing), as well as the roles that researchers play within health policy and systems (being) and in the conduct of HPSR itself (doing). We end by outlining questions researchers might use to deepen these three dimensions of their practice. The audience we address are all people engaged in conducting HPSR, working in government, NGO, or university settings with full-time or part-time research roles, who seek to support change in health policies and systems so as to improve people’s lives and address social inequalities.

## Seeing – systems with people at the core

### Health systems operate in broader contexts that are strongly influenced by people

There is an emerging appreciation of the evidence that shows that health systems are entrenched in their social, political, and economic settings, responding to health needs that themselves are generated by social, political, and economic forces and strongly shaped by the decisions and actions of historical and current day actors [8-10]. Broader contextual influences also seep into the daily practice of a health system through the experiences, mindsets, and values that shape the behaviours of the actors within it [11,12] (see Case study 1). As a result, despite similar elements and patterns, they can respond radically differently to the same new idea, policy, or intervention [13]. The dangers of HPSR that purports to support context-free and widely generalizable knowledge is increasingly evident [14], as also shown by the emerging social science-informed perspectives on paying for performance [15,16]. ‘Single truth’ research findings can distort holistic developmental agendas in low- and middle-income countries and divert resources towards solutions that are not appropriate to context, especially when there is very limited research on a particular issue. Fully understanding health systems in order to bring change within them therefore requires that HPSR explicitly accounts for context (Case studies 1 to 3).

### Health systems are constituted by people

Health systems have traditionally been conceptualized in instrumental or functional terms [17,18], but are now increasingly understood to have complex and variable attributes that are dynamically shaped by human agency at all levels (Case study 1). These include attributes of the so-called hardware of health systems – technical, financial, and material resources – that are shaped and driven by human choices and ingenuity; health system software – ideas, interests, values, norms, and relationships; and the interplay between the software and hardware [5,19]. As social institutions, it is people who ultimately determine the character of a health system. Health policy and systems researchers must seek better to understand these human qualities of health systems and policies – and their variations as well as similarities across varied settings (Case studies 1 and 2).

The people who make up health systems occupy various, sometimes multiple, positions – as providers, managers, financers, knowledge agents, and users of services. It is important to recognize that all these people are potential sources as well as users of valuable knowledge about the system, often moving between the two roles. Recognizing the useful knowledge held by health system actors allows health policy and systems researchers to access a greater breadth of such knowledge, including practical, moral, and cultural wisdom drawn from lived experiences and accumulated understanding of health system realities [20] (Case studies 1 to 3). Equally, people operating in different layers of the system (and not only designated policymakers) have diverse capabilities of processing and utilizing knowledge to bring about systemic improvements [21,22].

### Health policies and systems should serve people, especially the most vulnerable

Inequities in access to material and knowledge resources that improve health, and in the ability to influence important decisions, are a defining feature of health systems and policies (as they are of society at large). It is widely established that imbalances of power and social injustices underpin disparities in access to health resources, with particular communities, groups, and demographic categories in many countries often disproportionately experiencing poor health [23]. Any understanding of health systems must embrace an appreciation of power in and across systems and communities, and HPSR should have an active intention to address equity and social justice [24,25] (Case study 1). Such research should actively recognise the agency and capabilities of poor and socially marginalized people rather than reinforcing a view of them as passive recipients of policy.

***Case study 1: Ethnographic research on health workers in Koppal, southern India.*** *Private providers in poorly regulated health systems are at times seen as unrestrained in their profit-maximising motives, while government health workers can be characterised within a narrative of demoralisation and/or indifference*[26]. While both of these depictions are not entirely untrue, they occlude how health workers are also social actors making discerning choices in constrained circumstances, embedded in a myriad of social relationships that influence their personal and professional positions within specific political economies. Prolonged immersive ethnographic research in Koppal district, southern India, revealed various quid-pro-quo bargains struck between frontline health workers, other providers, and the communities they serve. Informal providers negotiated training and referral with formal providers in both government and private sectors, ensuring the filling of vacancies and continuity of services, even if at times laced with financial gains. Pressed for economic survival as salesmen in rural markets, they also depended on social ties with communities for their placement, reputation, and protection from the authorities. So while they felt forced to provide injections and tablets to demonstrate ‘strong’ treatment, they were at times inhibited from collecting debts from the communities they serve [27]. Female auxiliary health workers struggled for respect and recognition at various levels, starting within their homes. Yet, economically secure government positions also enabled them to leverage cooperation from their husbands and other family members. Auxiliary health workers actively sought and succeed at times in negotiating or stalling transfers, disciplinary memos, and other bureaucratic processes to ensure their interests. Rather than being passive bystanders, they develop coping mechanisms that are self-protective in hierarchical and at times vindictive accountability drives, even if they result in poor patient outcomes. The ethnographic approach, entailing repeated interactions over long periods that built trust and familiarity, and triangulation of interview data with observations of everyday events, developed an intimate account of these frontline providers. It enabled seeing the workers as active social agents, ingeniously navigating complex health systems, and thus provided alternative understandings of how service delivery outcomes are realised [27,28]. By creating more nuanced portraits of both positive and negative dimensions of frontline health workers and moving away from assumptions that either private or government workers are saviours or miscreants, creates the possibility of more informed policy responses that takes into consideration these incentives and their socio-political contexts.

## Being – locating the researcher in the health system

### The researcher in his/her (social, political, and economic) context

All those engaged in research and analysis on health policy and systems are part of the web of actors and organisations shaped by social, political, and economic forces that together make up health systems. Their choices of themes and questions, the way they approach them, the way they present research findings, and the power that they wield (and are, in turn, subjected to) are integral to the political economy of health policy and systems functioning. ‘Reflexivity’ is the bedrock principle of HPSR that must guide researchers in acknowledging and qualifying their own choices and positions vis-à-vis the research and analysis they undertake, and being explicit about their own interests, power, and relationships with policy processes and determinants of change [29]. In a value-laden science, explication by the researcher/analyst of their philosophical orientation and the broader goals of their research is also an important part of their reflexivity.

### The researcher’s relationship with other health system actors

**Figure 1:**
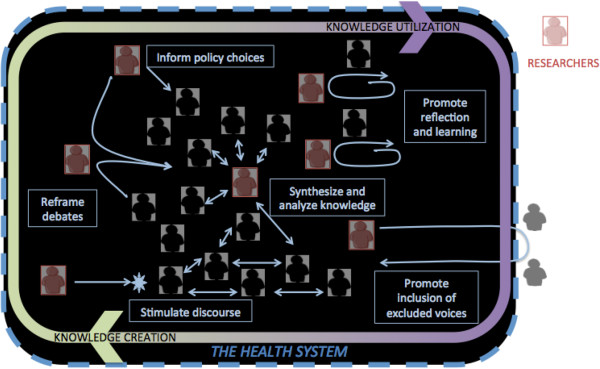
Health policy and systems research: a dialogic practice.

Health policy and systems researchers are themselves actors within, and an integral part of, health systems, but in conducting any form of research play a special role in aggregating, synthesising, and analysing available knowledge. A research practice that targets effective systemic change, as HPSR does, must be one that entails engagement with various actors within the system (Figure 1) (Case study 2) – moving well beyond the oft-invoked exhortation of ‘getting research into policy and practice’ that implies a schism between the researcher and the researched, and the worlds of knowledge and practice [30]. The quality and consistency of such engagement amplifies opportunities for the creation of strategic knowledge about the health system, as well as its uptake and utilization in strengthening health systems [1]. HPSR is always both an act and a product of dialogue, which takes place over time between those conducting research and other health system actors – with the latter acting formally as commissioners, collaborators and/or participants in research, or less formally as gatekeepers of health system information, informal informants, and brokers and users of research findings [31]. However, a research practice built on interaction and dialogue with stakeholders can raise challenges for researcher probity, which calls for the evolution of distinct ethical norms and practices for HPSR, with special attention to issues of conflict of interest.

Engagement with others conducting HPSR is also a key facet of a dialogic practice, with utility in terms of building alliances, and for positioning and fine-tuning research. Membership of a research and practice community offers added benefits such as opportunities for capacity building and peer support and review.

### Contractor or change-agent?

Even as health policy and systems researchers engage with decision-making it is important that they are not constrained to act as contracted purveyors of information to direct seemingly simple policy choices (whether by government decision-makers or funding agencies) [32]. Health policy and systems researchers have a far broader, and potentially more transformative role in the system, necessitating autonomy in the development of ideas and in practice. They can engage in generating new ideas for policy, reframing policy debates to make them more useful or more ethical, and ensuring that under-represented groups are heard [33]. Researchers can also crucially play a role in policy learning, by engaging with other health system actors directly to support them to reflect on and rethink existing problems (Case studies 2 and 3), or indirectly by stimulating public debate and opinion [31]. These transformative avenues entail varied forms of dialogue with a range of stakeholders (Figure 1).

***Case study 2: District-level action learning and reflective practice in South Africa and Kenya.*** Governance is often understood as a function of the formal, organisational centres of a health system (at both national and decentralised administration levels). Yet experiences of policy implementation identify the important influence of front line, and informal, governance processes, including community accountability, over implementation. In particular, the practices of health workers and front line managers have an important influence over the trajectory of policy change and health system development [34]. Full understanding of the nature of, influences over, and consequences of these practices requires engagement with the tacit knowledge of these health system actors and of the social situations and relationships within which they are embedded. A team of South African researchers initiated a long-term process of collaborative research with local health managers in one geographical area, based on a reflective practice and action learning approach [35]. This experience has also informed the development of two other local level ‘health system learning sites’ at district level in South Africa and Kenya. These collaborations are allowing inquiry into local level governance processes, including leadership and management practices, priority setting and problem-solving within primary health care facilities, and managing staff and relationships with actors from the community and other sectors. These processes are also supported in these collaborations as they provide opportunities for local actors, such as primary health care facility managers and their supervisors, to reflect on their work, develop new approaches to specific tasks, share and discuss their experience, and adapt and further develop these approaches. Learning, for example about the leadership and management capabilities needed at primary health care facility and sub-district levels, and about how to support leadership development at these levels, is occurring both as a result of more formal processes of inquiry and through cycles of collaborative reflection, action, and learning. This form of research engagement has provided spaces for trust-building between researchers and health system actors, and enabled rigorous knowledge generation, while simultaneously supporting action for health system strengthening.

## Doing – people centred research for real-world change

### Identifying topics and questions of relevance

The purpose of HPSR is to create useful knowledge about health policies and systems. Such knowledge includes capturing and explicating the complexity and interconnectedness of the various elements [36] in a way that has meaning to the people who make up the system and who can bring about appropriate systemic changes in different social contexts [37]. Such people include not only those at the upper echelons of power in their respective health systems, but also those at intermediary levels, care providers, users of services, and communities [21,22] (Case study 1 and 2). Close and consistent engagement with health system actors at different levels can enhance the relevance of HPSR topics and questions, and also the likelihood of use of the findings [38] (Case study 2).

### Conducting research of high quality

Since HPSR involves the use of methodologies that draw from different disciplines and knowledge paradigms, yardsticks and standards for research conduct vary in accordance with the methodology being applied [6,38]. Whilst statistical generalizability may be the gold standard for assessing the validity of claims drawn from quasi-experimental studies, analytic generalizability is the yardstick for case study research. However, quality in HPSR is a concern not only in applying the appropriate methodological rules to guide data collection and analysis, but at every step in the research endeavour – it is a holistic concern, ranging from how research questions are framed to address real-world concerns, to matching the question with the appropriate research approach and methodology, adapting methodology in response to real world realities, and the interpretation and utilization of research findings. Such quality is not necessarily (or solely) achieved by solitary thinking. In HPSR, as a dialogic science, quality is instead driven by engagement, contextualization, closeness to operational reality, attention to relevance, and reflexivity and the values that drive research practice [31]. Research questions need to be assessed to judge if they address issues that are relevant to the people and organisations trying to bring about positive change in systems (Case study 2). Too frequently, methodologies are matched with research questions without consideration of fit or appropriateness (as when structured survey instruments are the sole approach used to assess how health system actors understand particular experiences). Furthermore, as discussed earlier, context-free interpretation or application of research findings carries grave risks and dangers, and hence must also be regarded as an issue of quality.

### Policy-mindedness in research outputs

Policy-mindedness is a crucial attribute for health policy and systems researchers. They should not just aim to present information and evidence, but also to frame and situate findings and recommendations in actionable, rather than generic terms. What is actionable is subject to political viability, contextual appropriateness, and prevailing balances of policy priorities both within and outside the health sector (Case study 3). HPSR can only be enriched if researchers, regardless of which disciplinary framework they have applied, are attentive to the relevance of their findings for health and social policy in a given setting, as well as to the broader social and political contexts to which they pertain. The possibility of ‘evidence-driven’ but context-free research disrupting local priorities and development processes must be avoided. When there is limited formal health literature on the subject of enquiry, it does not necessarily imply an absence of ideas and experience on it. Frequently, discussions relevant to health policy and systems issues can be found in the literatures of related fields such as the social, policy and organisational sciences and sector-specific studies other than health, as well as in the media and in communities. Researchers can enhance the relevance of their findings by referencing discourses that lie outside the formal research literature (Case study 3). HPSR has an active place for descriptive and exploratory research, accessing such alternate discourses and unearthing tacit knowledge, especially in settings that are poorly explored and understood, such as in many low- and middle-income countries [4].

***Case study 3: Knowledge uptake for policy on community participation for health in India.*** In 2010, a high-level committee developing national recommendations on universal health coverage in India tasked a technical team of researchers with the synthesis of evidence on community participation for health (CPH). In the following year, several ‘learning cycles’ took place involving the committee and the technical team, until recommendations were developed [39].The technical team commenced with a structured review of the global peer-reviewed literature on CPH. When presented to the expert committee, some global norms and practices supported through research and advocacy in high-impact journals, were felt to be debatable when viewed in context in India. For instance, the question of ‘piece-rate’ or task-based incentives for frontline health workers [40], was deliberated. Discussions highlighted that, in spite of advantages, relying on piece-rate payments could reinforce selective caregiving and a medicalized view of community health – at odds with the long-term and comprehensive role of community health champion and mobilizer envisioned for India’s ASHAs in a universal health coverage milieu. The committee eventually recommended a basic fixed emolument supplemented by limited performance incentives.Major evidence gaps also diminished the usefulness of the initial review, with its exclusive reliance on evidence from journals. For instance, formal evidence on the significance of decentralized governance for improved community health is scarce – yet the Panchayati Raj system of local self-governance is integral to the overall impetus of social sector reforms in India, and hence found a prominent place in the report.The technical team expanded the knowledge-base to include first-hand consultations with informants from 20 organizations engaged with CPH on the frontlines and review of informal ‘grey’ reports of CPH interventions in India. Influenced by principles of realist synthesis [41] and analytic generalization [6], the technical team also refined their approach to analysing available knowledge, focusing on delineating ‘mechanisms’ that underpin successful interventions. Examples of CPH mechanisms that passed the dual test of evidence-support and local credibility (as assessed by the expert committee) included NGO roles in training and strengthening government-community relations; community health worker mentorship and support networks; and sustained financial and institutional support for capacity building for CPH – these informed the eventual recommendations. The knowledge uptake process, reflecting overlapping, iterative cycles of collaborative learning, benefited from consistent dialogue between the committee and technical team.

## Conclusions

We have argued that the guiding principles of a people-centred and change-oriented practice of HPSR inherently entail understanding the human and people-defined attributes of health systems more closely (seeing), actively considering the relationship of the researcher to the research (being), and working with an understanding of research quality that embraces context and relevance (doing). We conclude by proposing a set of questions across these three dimensions that health policy and systems researchers may wish to consider in making their practice more people-centred, and hence more oriented toward real-world change:

### Seeing: health systems with people at the core

•Does the research explicitly address dynamism and complexity, allowing for the social, political, and economic drivers of human behaviour?

•Have I situated the topic and findings in their immediate policy contexts, and in their broader social/political contexts?

•Does my research design advance understanding of the human attributes (choices, needs, preferences, interests, power, values, etc.) of the health system/policies in the setting of the research?

•Does my approach to the health system/health policy allow for power, equity, and justice?

### Being: considering the researcher’s position

•Have I considered my intentions for undertaking the research, and my position vis-à-vis the research subject?

•Have I explicated my own position vis-à-vis my power, my influences, and my interests related to the research, and my value basis, as indicated by my philosophical position and the change I wish to see?

•Have I engaged with other researchers investigating this theme? What do I contribute to, and gain from being part of, the community of health policy and systems researchers?

•How do I, as a researcher, engage with other health policy and systems actors, on what terms and with what consequences for my research and for effecting change in health policy and systems?

•Do I have autonomy in developing my own research ideas and conducting my research?

•Does my research hold a transformative intention, beyond informing the choices of designated policymakers? Do I have a strategy for actualizing such transformative change?

### Doing: relevant, high quality research

•Who is my research for? Who do I see as the primary users of my research findings?

•How has my research been influenced, directly or indirectly, by the experiences and perspectives of health system actors (including service users and communities)? Which health system actors? Have I engaged, directly or indirectly, with any of them in the process of defining my thematic focus and research questions, and while interpreting findings?

•Is my choice of research approach matched to the research question?

•Does my research design and analysis approach apply parameters of rigour and quality that are appropriate to the methodology used?

•(Especially if the subject/setting is previously poorly explored) Does my research design incorporate the exploration of non-formal publications and relevant social and political discourse pertaining to the setting(s) in which my research is likely to be utilized?

•Have I considered the consequences of the interpretation or application of my research findings in the settings in which they are likely to be used? Have I considered the intended and unintended effects on other social and policy agendas?

## Competing interests

The authors declare that they have no competing interests.

## Authors’ contributions

KS, AG, and LG conceived of the article. KS wrote the first draft. KS, AG, and LG jointly revised the manuscript critically for important intellectual content and gave final approval of the version to be published.
